# Relapsing and refractory peritoneal dialysis peritonitis caused by *Corynebacterium amycolatum*

**DOI:** 10.1007/s00467-022-05801-0

**Published:** 2022-11-10

**Authors:** Shameer M. Habeeb, Haneen Yamin, Eva Simkova, Hazem S. Awad, Entesar A. Alhammadi, Loai A. Eid, Rubina Lone, Martin Bitzan

**Affiliations:** 1 Kidney Centre of Excellence, Al Jalila Children’s Hospital, Dubai, UAE; 2 Dubai Medical College, Dubai, UAE; 3 Mohammed Bin Rashid University of Medicine and Health Sciences, Dubai, UAE; 4 Laboratory Medicine, Al Jalila Children’s Hospital, Dubai, UAE

**Keywords:** Corynebacterium amycolatum, Pediatric peritoneal dialysis, PD peritonitis, Bacterial biofilm

## Abstract

**Background:**

Peritonitis is an important complication and cause of morbidity in patients undergoing peritoneal dialysis (PD). *Corynebacterium species*, often considered skin and mucosal contaminants, are a rare cause of PD-associated peritonitis and have been acknowledged in published guidelines for the diagnosis and treatment of PD peritonitis only over the last decade.

**Case-Diagnosis/Treatment:**

We present two children with difficult-to-treat episodes of PD peritonitis due to *Corynebacterium amycolatum*. Episodes were associated with fever, abdominal pain and cloudy dialysate, high dialysate polymorphonuclear leukocyte counts, and elevated serum C-reactive protein and procalcitonin concentrations. Symptoms persisted beyond 5 days in 4 of 5 peritonitis episodes, and peritonitis relapsed despite in vitro sensitivity of the bacterial isolates to guideline-recommended antibiotics. *C. amycolatum* was cultured from the PD catheter tip despite 4 weeks of intraperitoneal glycopeptide therapy and clinical peritonitis resolution suggestive of efficient biofilm formation. Our systematic literature search identified three previous (adult) case descriptions of *C. amycolatum* peritonitis, all with repeat episodes by the same organism. The incidence of *C. amycolatum* as a cause of PD peritonitis has not yet been established but is likely underreported due to challenges in species differentiation.

**Conclusions:**

*C. amycolatum* is a rarely identified cause of refractory and/or relapsing PD peritonitis. Species differentiation of non-diphtheriae *Corynebacterium* isolates is critical, and prolonged antibiotic treatment, preferably with a glycopeptide antibiotic, is recommended, with a low threshold for PD catheter change or removal in case of repeat peritonitis.

## Introduction

Peritonitis represents an important complication in patients receiving chronic peritoneal dialysis (PD) leading to hospitalization, peritoneal membrane failure, PD catheter loss, and change of dialysis modality [1]. Corynebacteria are Gram-positive, facultatively anaerobic, nonsporulating, generally non-motile rods [2]. Non-diphtheriae (coryneform) *Corynebacterium species* belong to the physiological flora of human skin and mucous membranes [2]. Species level differentiation can be challenging, especially between *C. striatum*, *C. amycolatum*, and *C. xerosis* [2, 3]. *C. amycolatum*, a non-lipophilic *Corynebacterium* that lacks detectable mycolic acids found in the remainder of Corynebacteria [2], is now considered a common opportunistic pathogen in humans [2, 4]. Antibiotic sensitivity of clinical isolates is variable, but all strains are susceptible to glycopeptides [2].

To date, only three cases of PD peritonitis due to *C. amycolatum* have been reported, all in adults [5–7]. Here, we present two children with several episodes of *C. amycolatum* peritonitis highlighting therapeutic challenges and the growing importance of this organism.

Peritonitis definitions are from the current International Society of Peritoneal Dialysis (ISPD) guidelines [8]. Peritoneal effluent for cell count and differentiation and for microbial culture was obtained according to standard recommendations [8, 9]. “Day 1” is defined as the date of clinical diagnosis, when effluent dialysate was sent for microscopy and culture. Empirical peritonitis treatment consisted of intraperitoneal (IP) cefepime or the combination of IP ceftazidime and vancomycin [9], in addition to heparin 250–500 units/L and oral fluconazole.

## Case 1

The patient was a 4-year-old boy with CKD stage 5D secondary to congenital bilateral kidney hypodysplasia. He commenced chronic automated PD (APD) at the age of 2 years. A first peritonitis due to *Acinetobacter baumannii* 1 year after PD initiation was successfully treated with IP antibiotics. A year later, he presented with intermittent abdominal pain over 2 days and cloudy dialysate effluent. The peritoneal effluent white blood cell (WBC) count was 308/µL (36.1% neutrophils). Gram stain and culture of the effluent dialysis remained negative after 5 days of incubation. Peripheral WBC was 13.4 × 10^9^/L and C-reactive protein (CRP) 54.9 mg/L (normal < 5 mg/L). He was discharged and observed closely. A week later, he presented with scrotal pain, sluggish peritoneal drainage, and peripheral edema. Ultrasound showed a strangulated inguinal hernia prompting emergency herniotomy. Perioperatively, he received a dose of intravenous (IV) ceftriaxone. He resumed PD 2 days after surgery but returned the same evening with fever and severe, diffuse abdominal tenderness. PD catheter tunnel/exit site and herniotomy incision were intact. Microscopy of the dialysate revealed 482 WBC/µL. Serum CRP (59.1 mg/L) and procalcitonin (3.67 ng/mL, normal < 0.05 ng/mL) were elevated, and IP treatment with cefepime was started. Pre-treatment dialysate effluent (50 mL) was used to inoculate aerobic and anaerobic blood culture bottles for enrichment (5 mL each). The remainder of the effluent was centrifuged, and the sediment directly plated on various media, including chocolate and fastidious anaerobic agar. The dialysate showed Gram-positive rods with numerous WBC. Blood culture bottles flagged positive after 24 h of incubation. Subcultures, plated directly on blood and chocolate agar, yielded growth of small gray, flat colonies after 48 h of incubation. They were identified as *C. amycolatum* using the VITEK 2 ANC (Anaerobic and Corynebacterium) Identification Card (BioMérieux), confirmed by matrix-assisted laser desorption/ionization-time of flight mass spectrometry (MALDI-TOF; National Reference Laboratory, UAE). Isolates were in vitro susceptible to penicillin, cephalosporins, clindamycin, and glycopeptides and resistant to trimethoprim/sulfamethoxazole (TMP/SMX) using Clinical and Laboratory Standards Institute (CLSI M45) guidelines [10]. Repeat dialysate effluent cultures on days 2 and 9 were sterile, and antibiotic treatment was discontinued after a total of 3 weeks.

The patient returned 2 days later with new effluent drain pain and dialysate pleocytosis (2,844 WBC/µL, 54% neutrophils). Treatment was restarted with IP cefepime. Effluent culture again yielded *C. amycolatum*. Because the effluent failed to clear, IP teicoplanin was added for a total antibiotic treatment duration of 4 weeks. Repeat effluent analyses were normal, and cultures remained negative following the completion of antimicrobial therapy (Table 1).

**Table 1:** Clinical and laboratory findings at *C. amycolatum* peritonitis presentation

Case #	Peritonitis		Dialysate			Peripheral blood		Antimicrobial therapy
Episode	Day of peritonitis	Clinical findings	WBC [per µL] (neutrophils)	Microbial culture (time to result reporting)		WBC (ANC) [per nL]	CRP [< 5 mg/L] / PCT [< 0.05 ng/mL]	
1a	Day 1	Poor effluent, signs of peritonitis, fever	482 (56.6%)	*C. amycolatum* ^1^ (5 days)		9.4 (7.2)	59.1 / 3.67	IP cefepime D1–D21
	Day 2	Abdominal pain	19,791 (90.5%)	-		-	-	
1b	Day 1 (23) ^2^	Dialysate drain pain	2,844 (54%)	*C. amycolatum* (5 days)		10.2 (4.5)	-	IP cefepime D1–D31
	Day 6		2,854 (58.8%)	No growth		-	-	IP teicoplanin added D6–D31
	Day 8/9		192 (13.1%)	No growth		6.4	-	
1c	Day 1 (245)	Abdomen soft, not-tender	1,674 (41.4%)	Effluent and exit site: *C. amycolatum* (4 days)		10.3 (3.9)	1.6 / -	IP cefepime D1–D32
	Day 5	Fever, painful dialysis, poor UF	888 (47.1%)	Effluent: no growth Exit site & groin: *C. amycolatum*		-	-	IP teicoplanin added D5–D32
	Day 38/39	Kidney transplant	35 (8.5%)	Effluent: no growth PD catheter tip: *C. amycolatum*		-	-	IV cefazolin ^3^ PD catheter removed
2a	Day 1	Abdominal pain, fever, vomiting, low BP ^4^	2,161 (85%)	*C. amycolatum* ^1^ (6 days)		5.2 (1.8)	63.7 / 7.61	IP ceftazidime and vanco D1–D22
	Day 3	Continuous fever, hypotension ^4^	-	-		-	- / 12.20	IV meropenem added D3–D7
	Day 10		2,087 (93%)	No growth		-	- / 1.13	PO TMP/SMX added D10–D22
2b	Day 1 (43)	Abdominal pain	225 (94%)	No growth		6.6	31.7 / 1.5	IP ceftazidime & vanco D1–13
	Day 9	Continued abdominal pain	63 (51%)	-		9.6	61.5 / 3.91	TMP/SMX added D9–13
	Day 14	Continued abdominal pain	-	-		-	-	PD cath removed, HD IV ceftazidime & vanco

^1^All *C. amycolatum* isolates of patient 1 were sensitive in vitro to penicillin, cephalosporins (including cefepime), clindamycin, and vancomycin and resistant to trimethoprim/sulfamethoxazole. The isolate of patient 2 was sensitive to erythromycin, gentamicin, trimethoprim/sulfamethoxazole, and vancomycin and resistant to clindamycin. Meropenem was not tested due to the lack of validated breakpoints for the organism

^2^Presentation 2 days after completion of preceding antibiotic therapy (relapse; day 23 of first episode)

^3^Perioperative prophylaxis for kidney transplantation

^4^Onset of symptoms two days before presentation. Blood cultures remained negative. Admission to PICU on day 3 (for two day) due to arterial hypotension and suspected septicemia. See text for more details

*–* not done; *ANC*, absolute neutrophil count; *CRP*, C-reactive protein; *HD*, hemodialysis; *IP*, intraperitoneal; *IV*, intravenous; *PO*, per oral; *PCT*, procalcitonin; *PD*, peritoneal dialysis; *PMN*, polymorphonucleocytes; *TMP/SMX*, co-trimoxazole; *UF*, ultrafiltration; *vanco*, vancomycin; *WBC*, white blood cells;

Seven months later, the patient presented again with cloudy dialysate and fever. The effluent showed 1674/µL WBC (41% neutrophils), and empiric treatment was started with IP cefepime. *C. amycolatum* was isolated and teicoplanin added. Swabs from PD exit site and groin (but not from nose and throat) were positive for *C. amycolatum* with identical antibiotic sensitivities.

A week after treatment completion, the child received a deceased donor kidney transplant with standard perioperative cefazolin prophylaxis. The PD catheter was removed during the transplant surgery, and *C. amycolatum* was grown from its tip. There were no signs of peritonitis or bacteremia post-transplant (Table 1).

## Case 2

The patient was a 5-year-old girl with CKD stage 5D secondary to congenital nephrotic syndrome, APD since age 2 years. She had preceding peritonitis episodes due to *Staphylococcus epidermidis* and *Streptococcus viridans*. At the index episode, she presented with fever, vomiting, lower abdominal pain, and poor oral intake for the past 2 days. Clinical examination showed fever of 38.7 °C and mild abdominal tenderness. Peripheral WBC was normal, yet CRP and procalcitonin were significantly elevated. Dialysate effluent revealed 2161 WBC/μL (85% neutrophils), and peritonitis treatment was started with IP ceftazidime and vancomycin. She remained febrile over the next 48 h, accompanied by multiple hypotensive episodes and rising serum CRP and procalcitonin concentrations prompting fluid boluses, transfer to the pediatric intensive care unit due to suspected sepsis (days 3–5), and addition of IV meropenem. The latter was discontinued when the effluent culture result became available: *C. amycolatum*, in vitro sensitive to erythromycin, gentamicin, TMP/SMX, vancomycin; resistant to clindamycin. Oral TMP/SMX was added on day 10 because of persistent fever and high effluent WBC. The patient was discharged on day 11 against medical advice. IP and oral antibiotics were continued for a total of 3 weeks (Table 1).

She was readmitted with severe abdominal pain 3 weeks after discontinuation of the antibiotics, with 225 WBC/μL effluent (94% neutrophils). IP ceftazidime and vancomycin were restarted and continued at home. The dialysate culture remained negative. She returned on day 9 of the peritonitis relapse due to persistent abdominal pain, associated with rising inflammatory markers (Table 1). Oral TMP/SMX was again added without clinical improvement, prompting PD catheter removal and transfer to HD.

## Literature review

In a systemic PubMed and Google Scholar search without language restriction, we identified three previously published (adult) cases of *C. amycolatum* PD peritonitis (Table 2). In the first reported case of a 65-year-old woman, the isolate was sensitive in vitro to all antibiotics used. Peritonitis only resolved after switching to IP vancomycin. Repeat peritonitis 3.5 months after treatment completion led to PD catheter removal and IV vancomycin administration, followed by successful PD re-initiation. Identity of both *C. amycolatum* isolates was demonstrated by pulsed-field gel electrophoresis [5]. Sonmezer et al. described a 55-year-old patient who developed *C. amycolatum* peritonitis associated with increased peripheral WBC count and CRP [6]. Effluent dialysate cleared after switching from IP cefazolin and gentamicin to IP vancomycin, yet she returned with turbid effluent five days after discharge. The infection resolved following combined IP and IV vancomycin administration (see Table 2). The third patient was identified in a study evaluating the utility of a taurolidine/citrate/urokinase PD catheter “lock” in patients with frequent peritonitis. The treatment protocol consisted of IP vancomycin and an aminoglycoside for 14–21 days. One of six enrolled patients had multiple episodes of *C. amycolatum* peritonitis, but further clinical details are missing [7]. Ubaldi et al. assessed the frequency of *Corynebacterium* isolates in a medical microbiology laboratory over a 3-year period [11]. *Corynebacterium* was cultured from 31 PD catheter exit sites and six PD fluid samples. Four and two isolates, respectively, were identified as *C. amycolatum*, without demographic and clinical data.

**Table 2:** PD-associated peritonitis due to *C. amycolatum*. Summary of identified case reports

Case # Reference	Patient age (sex)	Primary renal disease	Time from start of PD to peritonitis ^1^	Peritonitis episodes (interval)	Treatment ^2^	Clinical outcome
# 1 [5]	65 y (F)	Chronic glomerulonephritis	5 months CAPD	2 (3.5 months) ^3^	(a) IP cefazolin and ceftazidime, followed by IP vancomycin (day 8) (b) PD Cath removal and IV vancomycin	Return to PD Peritonitis-free 6 months of observation
# 2 [6]	55 y (F)	Nephrotic syndrome	2 years CAPD	2 (4 days) ^4^	(a) IP cefazolin and gentamicin, followed by IP vancomycin (day 5) (b) IP and IV vancomycin	No further peritonitis reported
# 3 [7]	36 y (F)	-	2 years APD	9 (< 4 weeks)	-	-
# 4 This publication	4 y (M)	Congenital renal hypoplasia	45 months APD	3 (2 days) ^4^ (7 months) ^3^	(a) IP cefepime (b) IP cefepime and teicoplanin (c) IP cefepime and teicoplanin	PD cath removal at time of successful kidney transplant
# 5 This publication	5 y (F)	Congenital nephrotic syndrome	3 years APD	2 (3 weeks) ^5^	(a) IP ceftazidime and vancomycin IV meropenem (5 days) PO TMP/SMX (see text) (b) IP ceftazidime and vancomycin PO TMP/SMX (see text)	Relapsing peritonitis PD cath removal Transfer to HD

^1^Time from initiation of PD to first *C. amycolatum* peritonitis and PD modality

^2^All *C amycolatum* isolates were sensitive to the antibiotics used

^3^Repeat peritonitis (same organism, based on pulsed-field gel electrophoresis)

^4^Relapsing peritonitis

^5^Peritonitis relapse (culture-negative)

*–*, not reported; *APD*, automated peritoneal dialysis; *CAPD*, continuous ambulatory peritoneal dialysis; *TMP/SMX*, co-trimoxazole

## Discussion

*C. amycolatum* PD peritonitis has not yet been reported in the pediatric age group. Although rare, the true incidence may be greater than suggested by the current literature [11]. Species differentiation of non-diphtheriae *Corynebacterium* strains is technically demanding [2, 12], leading to delayed resulting and genus-level reporting only. For example, a recent registry study comprising 11,122 PD peritonitis episodes in adults attributed 162 episodes (1.5% of all peritonitis cases) to non-diphtheriae Corynebacteria. The authors found no difference in relevant outcome parameters, such as clearing of infection, PD catheter survival, or peritonitis-related death between episodes due to Corynebacteria or other Gram-positive organisms [13]. In contrast, an earlier study from Hong Kong reported increased rates of repeat peritonitis due to non-diphtheriae Corynebacteria [14]. Neither publication provided species level identification, which may have obscured differences in species-related outcomes. Species identification has clinical importance, allowing to separate chronic from de novo infections and to adjust antibiotic treatment or opt for PD catheter removal if the same strain is isolated repeatedly.

Based on these cases, a picture emerges of *C. amycolatum* as a cause of PD peritonitis with notable, occasionally severe systemic inflammation that tends to be refractory to conventional, guideline-based treatment and prone to repeat episodes even after prolonged periods of quiescence. Interestingly, effluent cultures became promptly negative after initiation of IP antibiotic therapy in our patients, yet dialysate pleocytosis and abdominal discomfort persisted in four of the five (refractory) episodes [8] (Table 1). Both patients experienced “relapsing” peritonitides, defined as occurrence within 4 weeks of treatment completion, even in the absence of bacterial growth [8] (Table 1, episode 2b). It is not primary antimicrobial resistance but PD catheter biofilm formation that appears to complicate conventional treatment of *C. amycolatum* peritonitis [7, 15]. Possible targets for research are the development of biofilm-disruptive therapies, understanding bacterial and host factors that facilitate biofilm formation, and strategies for its prevention.
